# Dopamine dysfunction in depression: application of texture analysis to dopamine transporter single-photon emission computed tomography imaging

**DOI:** 10.1038/s41398-022-02080-z

**Published:** 2022-08-03

**Authors:** Takehiro Tamura, Genichi Sugihara, Kyoji Okita, Yohei Mukai, Hiroshi Matsuda, Hiroki Shiwaku, Shunsuke Takagi, Hiromitsu Daisaki, Ukihide Tateishi, Hidehiko Takahashi

**Affiliations:** 1 Department of Psychiatry and Behavioral Neurosciences, Graduate School of Medical and Dental Sciences, Tokyo Medical and Dental University, Tokyo, Japan; 2 Department of Diagnostic Radiology and Nuclear Medicine, Graduate School of Medical and Dental Sciences, Tokyo Medical and Dental University, Tokyo, Japan; 3 Integrative Brain Imaging Center, National Center of Neurology and Psychiatry, Tokyo, Japan; 4 Department of Neurology, National Center Hospital, Parkinson’s Disease & Movement Disorders Center, National Center of Neurology and Psychiatry, Tokyo, Japan; 5 Department of Biofunctional Imaging, Fukushima Medical University, Fukushima, Japan; 6 Graduate School of Radiological Technology, Gunma Prefectural College of Health Science, Gunma, Japan

**Keywords:** Depression, Diagnostic markers

## Abstract

Dopamine dysfunction has been associated with depression. However, results of recent neuroimaging studies on dopamine transporter (DAT), which reflect the function of the dopaminergic system, are inconclusive. The aim of this study was to apply texture analysis, a novel method to extract information about the textural properties of images (e.g., coarseness), to single-photon emission computed tomography (SPECT) imaging in depression. We performed SPECT using ^123^I-ioflupane to measure DAT binding in 150 patients with major depressive disorder (*N* = 112) and bipolar disorder (*N* = 38). The texture features of DAT binding in subregions of the striatum were calculated. We evaluated the relationship between the texture feature values (coarseness, contrast, and busyness) and severity of depression, and then examined the effects of medication and diagnosis on such relationship. Furthermore, using the data from 40 healthy subjects, we examined the effects of age and sex on the texture feature values. The degree of busyness of the limbic region in the left striatum linked to the severity of depression (*p* = 0.0025). The post-hoc analysis revealed that this texture feature value was significantly higher in both the severe and non-severe depression groups than in the remission group (*p* = 0.001 and *p* = 0.028, respectively). This finding remained consistent after considering the effect of medication. The effects of age and sex in healthy individuals were not evident in this texture feature value. Our findings imply that the application of texture analysis to DAT-SPECT may provide a state-marker of depression.

## Introduction

The molecular basis of depression, including major depressive disorder (MDD) and bipolar disorder (BD), has not been fully investigated. Antidepressants, which primarily target the serotonin and noradrenaline systems, have been shown to be effective for the treatment of MDD, which leads to the monoamine hypothesis of MDD. However, it has been found that 70% of patients with MDD do not achieve remission with antidepressant treatment [1]. In particular, the presence of pleasure loss, one of the core symptoms of MDD, has predicted inadequate response to antidepressants [2–4]. Loss of pleasure, along with decreased motivation, has been associated with dopamine hypofunction in MDD [5]. Alterations in dopamine function in BD have also been associated with its pathophysiology. Specifically, it is hypothesized that opposing changes in dopaminergic function are observed in manic and depressive states and that the disruption of the dopamine receptor and transporter homeostasis might underlie these changes [6].

Dopamine dysfunction in depression has been supported by recent neuroimaging studies investigating the dopamine transporter (DAT) [7–10]. DAT plays an important role in dopamine regulation at the synapse. It is a plasma membrane protein selectively expressed in dopaminergic neurons [11]. DAT binding reflects the number of presynaptic dopaminergic neurons as well as the amount of DAT at presynaptic terminals in each dopaminergic neuron [7] based on imaging studies. Several studies have established that depression in MDD is related with the reduced binding of DAT in the midbrain [8] and striatum [7, 9], although the results of DAT imaging in bipolar depression have been inconsistent [6]. Reduced DAT binding is a possible underlying mechanism in depression that is postulated to be the downregulation of DAT caused by chronic dopamine depletion [12, 13]. However, it remains unclear whether it depends on the diagnosis of depression or on the state of depression. DAT single-photon emission computed tomography (SPECT) in patients with MDD had shown inconsistent results on two previous longitudinal studies. One study showed a significant increase in DAT binding in the midbrain in the group with improved depression compared with the group without improved depression [14], whereas the other showed no significant correlation between DAT binding and the change in depression scores with treatment [15]. Moreover, it has been suggested that the duration of illness, number of major depressive episodes (MDEs) and receiving electroconvulsive therapy (ECT) may affect the DAT binding in the striatum, although previous positron emission tomography (PET) studies have shown inconsistent results [7, 9, 16].

The distribution volume ratio (DVR) was employed by neuroimaging studies as an index derived from mean uptake analysis of a region of interest (ROI) in the striatum, as a standard measure of DAT binding [17]. This index indicates the density of DAT in a ROI, and DVRs obtained from DAT-SPECT and PET correlates well across resolution differences [18]. However, this conventional analysis tends to excessively simplify the available spatial information [19]. Thus, novel approaches have been introduced to extract features from images to quantify tissue properties. Texture analysis is an example, which can yield features for quantifying intuitive image textures, such as coarseness, contrast, and busyness [20]. Features derived from texture analysis provide three-dimensional information regarding the heterogeneity of uptake in a tissue, including the intensity histogram of the image and relative position of pixels/voxels [19, 21]. Because texture features are calculated based on the spatial information, the features could provide more clinically important information. In fact, a study which applied texture analysis to the DAT-SPECT images of Parkinson’s disease (PD) reported that texture features correlated with clinical measures, whereas conventional DVR did not [19]. The results are in line with those of a postmortem brain study, which showed that dopamine loss in the striatum occurs with high spatial heterogeneity in PD pathology [22]. These findings imply that the alterations in DAT could be detected in further detail by applying texture analysis to DAT imaging in depression.

In the present study, we conducted texture analysis to DAT-SPECT in patients with MDD and BD. This technique has not yet been applied to DAT-SPECT images in depression. We hypothesized that the texture features in the striatum derived from DAT-SPECT would be altered according to the severity and phase of the disorder. To the best of our knowledge, this is the first study to apply texture analysis to depression with the sample size of 150. Moreover, a healthy group of 40 individuals was evaluated to examine the effects of normal aging and sex on the texture features. We then explored the texture features of DAT-SPECT that could better depict the dopamine dysfunction in a depressive state compared with conventional ROI analyses.

## Materials and methods

### Subjects

We included the data from patients who underwent DAT-SPECT at the Department of Psychiatry, Tokyo Medical and Dental University Hospital between April 2016 and October 2021 who were diagnosed with MDD and BD based on the Diagnostic and Statistical Manual of Mental Disorders criteria [23]. Data from a total of 150 patients (112 with MDD and 38 with BD) was included and extracted after selection based on the following exclusion criteria: (1) a diagnosis of Lewy body disease (LBD; PD and dementia with Lewy bodies) or PD-related diseases (multiple system atrophy, progressive supranuclear palsy, and corticobasal ganglia degeneration), (2) suspected LBD or PD-related disease for which dopamine replacement therapy was clinically indicated by a neurologist (3) a diagnosis of substance-related disorders, and (4) a diagnosis of other neurological diseases, such as extensive cerebrovascular disease and brain tumor. Clinical information was obtained, including sex, age, severity of depression assessed using the Hamilton Depression Rating Scale (17 items) (HDRS17) [24], the duration of illness (months), the total number of MDEs, and the history of ECT with one year. Also included was information on smoking[25] and medications that is, tricyclic antidepressants, selective serotonin reuptake inhibitors (SSRIs), serotonin and noradrenaline reuptake inhibitors (SNRIs), and memantine, which may affect the values in DAT-SPECT. Furthermore, to examine the longitudinal change in the course of depression in the same individuals, we analyzed the data of the phase of depression (pre-treatment) and remission (post-treatment) in a subset of the sample (*N* = 12). These patient data are unpublished. To evaluate the effects of age and sex on the texture feature values, we also analyzed the images of 40 healthy subjects that were used in a previous study that was approved by the Ethics and Review Board of the National Center for Neurology and Psychiatry, Tokyo, Japan (A2014-125) [26]. All participants were Japanese. The study protocol was approved by the Ethics and Review Board of Tokyo Medical and Dental University Hospital, Tokyo, Japan (M2019-038). Consent of the participants was obtained using the opt-out method since this study was conducted using only medical records.

### Image acquisition and processing

#### DAT-SPECT images

The subjects were imaged 3 h following the injection of approximately 167 MBq of ^123^I-ioflupane that is, ^123^iodine-labeled N-(3-fluoropropyl)-2β-carbomethoxy-3β-(4-iodophenyl) nortropane (^123^I-FP-CIT). In the patient group, the ^123^I-FP-CIT SPECT images were acquired using the Symbia E scanner (Siemens Healthineers, Erlangen, Germany). The tomographic projection images were acquired in continuous mode over 360°, a zoom of 1.00, and an image matrix of 128 × 128 pixels that resulted in a 1.72 mm × 1.72 mm × 1.72 mm voxel size. Projection images were reconstructed with the ordered subset expectation maximization (OS-EM) algorithm. No scanner recalibration had been performed within the study period. In the healthy group, the tomographic projection images were acquired in continuous mode over 360°, a zoom of 1.45, and an image matrix of 128 × 128 pixels that resulted in a 3.3 mm × 3.3 mm × 3.3 mm voxel size using Symbia T6 (Siemens Healthineers). Subsequent processing was the same as in the patient group.

#### Magnetic resonance (MR) images

The data of the MR images were collected using the 3-Tesla MAGNETOM spectra (Siemens Healthineers). The parameters of the acquisition were summarized in Table S1. T1-weighted MR images were acquired to determine the anatomical brain structure.

#### ROI creation

**Fig. 1:**
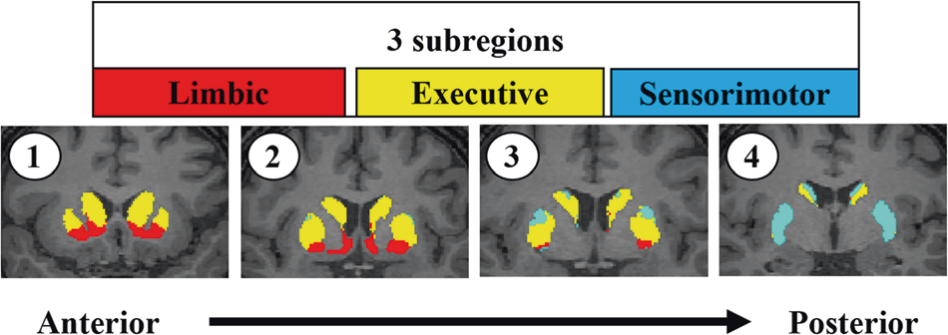
Region of interest in the striatum. Striatal atlas reflecting the probabilistic anatomical connections between the cortex and the striatum, divided into three subregions. Each voxel in the striatum was classified according to the cortical region with the highest connection probability. These probabilities are defined as the number of subjects with connection probabilities of >25% divided by the total number of subjects.

We first converted the voxel size of the MR images into 1 mm × 1 mm × 1 mm, and the SPECT images were co-registered to MR images to convert the same voxel size and coordinate system. We segmented the MR images to obtain the boundaries of the striatum utilizing the Oxford-GSK-Imanova Striatal Connectivity Atlas [27], as well as the occipital cortex as a reference region using the Automated Anatomical Labeling Atlas [28], adapted to the individual brain. The Oxford-GSK-Imanova Striatal Connectivity Atlas segmented the striatum into three subregions (i.e., limbic, executive, and sensorimotor regions; Fig. 1). We conducted these processes using SPM12 (Functional Imaging Laboratory, UCL) running on MATLAB (MathWorks).

#### Extraction of features using texture analysis

We selected the Neighborhood Gray Level Difference Matrix (NGLDM) among the texture feature types, because the matrix had been shown to have a high-discriminative power between malignant and benign lesions [29]. We extracted three texture features based on the NGLDM (i.e., coarseness, contrast, and busyness), which have been described in detail in a previous study [20]. Briefly, coarseness is the most fundamental property and is indicated by the reciprocal of the sum of the difference between the gray tone of one pixel and the average gray tone of the neighboring surrounding pixels, weighted by the probability of occurrence of each intensity value. The level of spatial rate of change of intensity is indicated with large values indicating areas with small differences in gray tones that is, coarse textures [20]. Contrast is high when the intensity differences in the neighboring areas are large, which is influenced by the dynamic range of the gray scale and spatial frequency of changes in intensity. Busy texture is characterized as a very high spatial frequency of changes in intensity. The degree of busyness is indicated by suppressing the contrast aspect from the information of the spatial rate of changes in intensity. The numerator specifically indicates the level of spatial rate of change, the denominator is the sum of the magnitudes of differences between the different gray tone values, and each value is weighted by its probability of occurrence.

To calculate the texture features, the DAT-SPECT images were processed as follows. The uptake values contained within the ROIs were resampled prior to texture feature extraction using a 64-bin discretization between the minimum and maximum uptake values in the ROI. We normalized the voxel size and the number of gray levels before feature extraction to reduce the dependency of the texture features on these elements, which may affect several texture features [30]. The voxel size to be used to calculate the texture feature values in the ROI was set in 1 mm × 1 mm × 1 mm. The LIFEx software (ver. 7) (http://www.lifexsoft.org) [31] was used to calculate the texture features.

#### Extraction of conventional measures

We subsequently computed the mean radiotracer uptake values in each ROI, and divided them by those in the reference region to obtain an approximate estimate of the DVR. This was conventionally used as a quantitative outcome [17].

### Statistical analyses

The demographic variables were appropriately compared using Pearson’s χ^2^ test, Mann–Whitney U test, and Kruskal–Wallis test. The texture feature values obtained in each ROI were compared between the groups classified by the severity of depression using the Jonckheere–Terpstra test. Post hoc analyses were performed via Dunn’s test [32] using overall rank. Based on a previous study on DAT in depression [7], the patients were classified into three groups according to the HDRS17 score: remission (score ranging from 0 to 7), non-severe depression (score ranging from 8 to 18), and severe depression (score ≥19). Evaluation of intergroup differences in age, sex, number of MDEs, or the duration of illness were present. The following additional tests were performed only for those feature values that showed a significant F with the severity of depression. First, to test the effects of medication on the outcomes, we divided the patients into two groups: (1) patients under medication (e.g., SSRIs and SNRIs) that might affect the results of DAT-SPECT and (2) patients who were not under medication and underwent the same analyses. Second, to investigate the effects of diagnosis, the same analysis was performed in patients with MDD and BD, respectively. Third, to examine the effects of recent smoking or ECT that might affect the results of DAT-SPECT, the same analysis was repeated in patients who had no smoking history in the previous year or had no receiving ECT in the previous year. Fourth, to test whether the findings were observed in the course of depression in the same individuals, the data of the pre- and post-treatment phases were analyzed using Wilcoxon’s signed-rank test. Finally, to test the current consensus that dopamine dysfunction in depression is especially related to the symptom of loss of pleasure [5], we evaluated the relationships between the texture feature values and the severity of loss of pleasure, as well as depressive mood. In the analyses, the subscales of HDRS17 were used to divide the patients into three groups with no symptoms (score 0), non-severe depression (scores 1 and 2), and severe depression (scores 3 and 4), respectively. Three NGLDM texture features were calculated for each of the three bilateral regions (i.e., limbic, executive, and sensorimotor), and thus a total of 18 indices were evaluated. The Bonferroni correction for multiple comparisons was therefore applied with a *p* value of <0.0027 (0.05/18). We repeated the same statistical analyses on the DVR values (a total of 6 indices) and thus set a statistical significance threshold of 0.083 (0.05/6). We examined the effects of age and sex on the texture feature values using Pearson’s correlation in the healthy group. We performed statistical analyses using the SPSS 28 (IBM Corp., Armonk, NY, USA).

## Results

A total of 112 patients with MDD and 38 patients with BD were evaluated (Table 1). Both groups had a high percentage of females, and no significant difference in sex (*p* = 0.445) or severity of depression (*p* = 0.484) was observed between the diagnostic groups. However, differences in age, duration of illness, and the number of MDEs between the two groups (all *p* < 0.001) were observed.

**Table 1:** Clinical characteristics of the participants.

Clinical characteristics of the participants	MDD (*n* = 112)	BD (*n* = 38)	Statistics	*p* value	All patients (*n* = 150)	Healthy (*n* = 40)
Age, years, mean (SD, range)	71.4 (9.9, 41–89)	61.5 (15.5, 24–86)	U = 1327.0	<0.001*	68.9 (12.3, 24–89)	53.9 (13.7, 30–81)
Sex, ratio (male/female)	0.28 (31/81)	0.34 (13/25)	χ^2^ = 0.584	0.445	0.29 (44/106)	0.48 (19/21)
Smoking history in the previous year, No. (%)	11 (9.8)	6 (15.8)	χ^2^ = 1.006	0.316	17 (11.3)	N/A
Duration from the first MDE, months, mean (SD)	130.4 (143.9)	218.6 (166.6)	U = 1334.0	<0.001*	152.4 (154.2)	0
Lifetime MDE, No. mean (SD)	2.6 (2.2)	5.2 (2.9)	U = 918.5	<0.001*	3.3 (2.7)	0
Receiving ECT in the previous year, No. (%)	17 (15.2)	2 (5.3)	χ^2^ = 2.522	0.112	19 (12.7)	0
Medication which may affect the values in DAT-SPECT, No.						
TCA	4	0			4	0
SSRI	41	3			44	0
SNRI	32	3			35	0
Memantine	2	0			2	0
Two or more of the above drugs	7	1			8	0
Unmedicated	26	31			57	40
Severity of depression (based on HRDS17)			χ^2^ = 1.451	0.484		
Remission (0–7)	44	14			58	N/A
Non-severe depression (8–18)	39	17			36	N/A
Severe depression (≥19)	29	7			36	N/A

All of the patients were stratified into three groups according to the severity of depression using the Hamilton Depression Rating Scale (17 items) (HDRS17): remission group (score ranging from 0 to 7), non-severe depression group (8 to 18), and severe depression group (≥19).

*MDD* major depressive disorder, *BD* bipolar disorder, *SD* standard deviation, *MDE* major depressive episode, *ECT* electroconvulsive therapy, *DAT-SPECT* dopamine transporter single-photon emission computed tomography, *TCA* tricyclic antidepressant, *SSRI* selective serotonin reuptake inhibitor, *SNRI* serotonin and noradrenaline reuptake inhibitor, *N/A* not available.

*p < 0.05.

### Texture feature values in the patient group

Prior to the following analysis, we confirmed no significant difference in age, sex, smoking history in the previous year, the duration of illness, the number of MDEs, or receiving ECT in the previous year among the three groups based on the severity of depression (*p* = 0.091, *p* = 0.843, *p* = 0.157, *p* = 0.163, *p* = 0.078, and *p* = 0.072, respectively).

Four bilateral regions for three NGLDM texture features were investigated and we found intergroup differences in 5 out of 18 indices between the three groups. However, the feature value that survived the Bonferroni correction (*p* < 0.0027) was only busyness of the limbic region in the left striatum (busyness-LL) (*p* = 0.0025) (Table 2).

**Table 2:** Texture feature values and the severity of depression.

Texture feature values	*p* value					
	Limbic region		Executive region		Sensorimotor region	
	Left	Right	Left	Right	Left	Right
**Coarseness**	0.027*	0.005*	0.015*	0.466	0.055	0.066
**Contrast**	0.131	0.017*	0.207	0.593	0.229	0.131
**Busyness**	0.0025**	0.141	0.862	0.956	0.424	0.108

The texture feature values were compared between the groups classified by the severity of depression using the Jonckheere–Terpstra test. The Bonferroni correction for multiple comparisons was applied with a *p* value of <0.0027 (0.05/18).

*p < 0.05. **p < 0.0027.

**Fig. 2:**
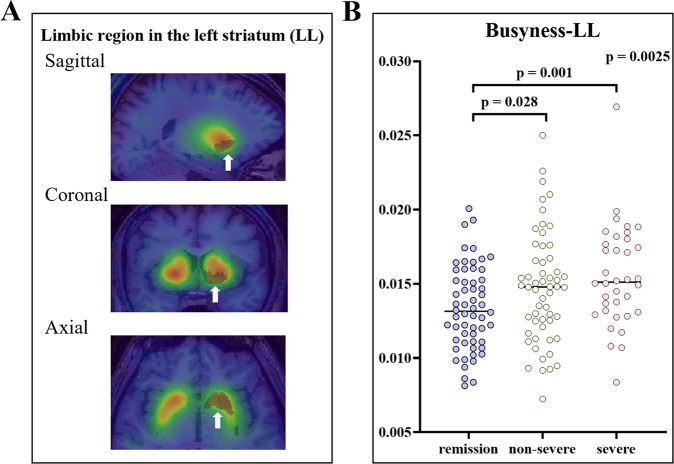
Busyness-LL and the severity of depression. **A** ROI of the limbic region in the left striatum superimposed on MR and SPCET images of a subject. Arrows indicate the ROI. **B** The degree of busyness-LL linked to the severity of depression. LL limbic region in the left striatum, ROI region of interest, MR magnetic resonance, SPECTs single-photon emission computed tomography.

The post hoc analysis showed the severe depression group had a higher busyness-LL than the remission group (*p* = 0.001), as well as the non-severe depression group (*p* = 0.028), but no significant difference was observed between the severe and non-severe depression groups (*p* = 0.133) (Fig. 2). We highlighted the busyness-LL in the subsequent validation analyses based on this finding.

**Fig. 3:**
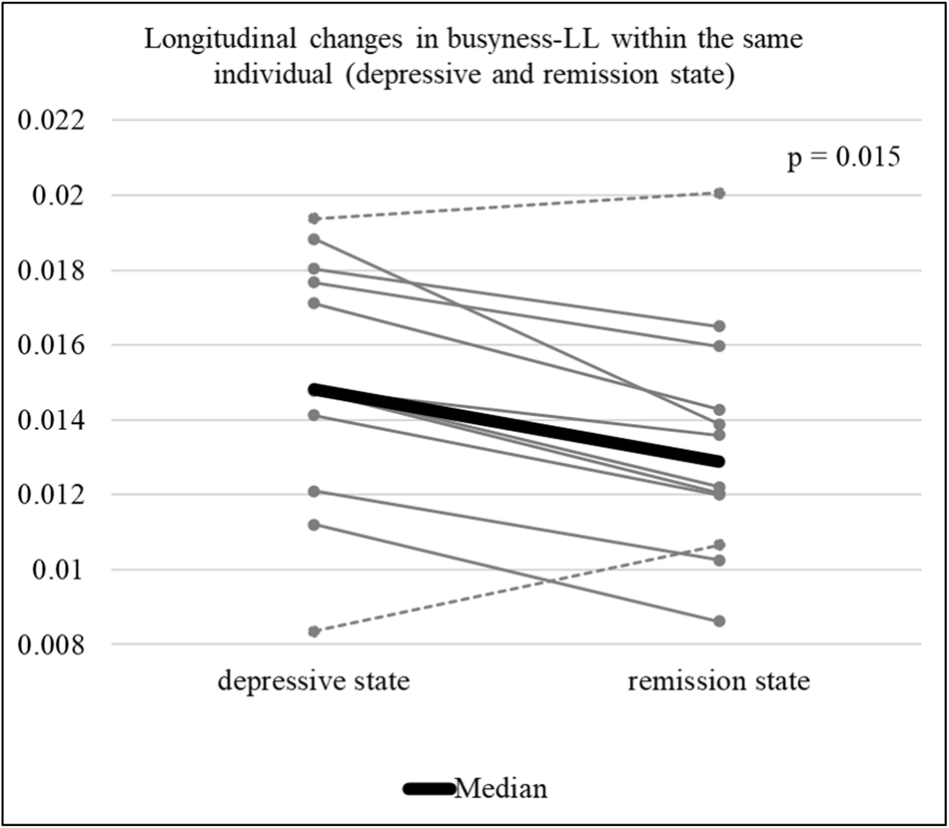
Longitudinal changes (depressive state [pre-treatment] and remission state [post-treatment]) in busyness-LL within the same individual (*N* = 12). LL limbic region in the left striatum.

We examined the effect of medication on busyness-LL in DAT-SPECT. In the medicated group (*N* = 93), a significant intergroup difference in busyness-LL was observed compared with the other groups based on the severity of depression (*p* = 0.008). In the unmedicated group (*N* = 57), the same trend, but not significant (*p* = 0.066), was observed (Fig. S1). Regarding the diagnostic effects, the same result was obtained when the subjects were limited to MDD (*N* = 112; *p* = 0.005), but no significant trends were found when the subjects were limited to BD (*N* = 38; *p* = 0.235) (Fig. S2). In addition, the result remained same when the subjects were limited to those who had no smoking history in the previous year (*N* = 133; *p* = 0.006) or had not received ECT in the previous year (*N* = 131; *p* = 0.002; Figs. S3, S4). The longitudinal changes in the same subjects with MDD with successful treatment showed that 10 out of 12 cases had the same trend as the overall results; busyness-LL was higher in the depressive state than in the remission state (*p* = 0.015; Fig. 3). Finally, we assessed the relationship between busyness-LL and the severity of the core symptoms of depression; depressive mood and loss of pleasure. Busyness-LL was significantly linked to the severity of both depressed mood and loss of pleasure (*p* = 0.033 and *p* = 0.003, respectively).

### Conventional DVR findings in the patient group

In the DVR values, the intergroup difference was not evident in each of the six indices. This remained the same when the analysis was limited to the medicated group (*N* = 93) and to the diagnosis of MDD (*N* = 112) and BD (*N* = 38). The unmedicated group (*N* = 57) showed significant differences in the DVR value of the sensorimotor region of the left striatum (DVR-SML) (*p* = 0.005) (Fig. S5). The longitudinal changes of DVR-SML showed that only 4 of 12 patients had the same trend as the overall results (Fig. S6).

### Effects of age and sex in texture feature values and DVR values in the healthy group

Tables S2 and S3 shows the effects of age and sex on the texture feature values in the healthy group. These results were not evident in busyness-LL but three texture feature values showed significant correlation with age and five texture feature values showed a significant difference between males and females In line with a previous study [33], the DVR values were found to be negatively correlated with age in the bilateral sensorimotor regions in the striatum (DVR-SML (*p* = 0.010) and DVR-SMR (*p* = 0.011)), whereas sex was found to have no significant effect on any of the DVR values.

## Discussion

In the present study, we showed that several feature values obtained from the texture analysis of DAT-SPECT were linked to the severity of depression in MDD and BD. Among the texture feature values, the most evident effect was found in busyness in the left ventral (i.e., limbic) region of the striatum (busyness-LL). This finding remained the same after considering the effect of medication and was statistically significant when the sample was limited to MDD. Furthermore, busyness-LL was strongly linked to the degree of pleasure loss. An analysis on a subset of the sample confirmed that this feature value was reversible according to a course of depression. In the healthy group, busyness-LL was found to be not affected by age and sex. In contrast, the conventional ROI analyses showed that the DVR values were not linked to the severity of depression and were affected by medication. Our findings suggest that the texture analysis of DAT imaging may provide feature values that can be a state-marker of depression.

Previous studies evaluating DAT binding in depression have yielded inconsistent results. Meta-analysis based on SPECT studies showed unchanged DAT binding in depression [34], whereas a series of recently published PET studies has reported a reduction in DAT binding in MDD [7–9]. This discrepancy is attributable to differences among the studies in several aspects including the differences in the severity of depression of the participants, medication status, and spatial resolutions between SPECT and PET. Our findings of altered DAT feature values obtained by texture analysis in depression may overall support the results of recent PET studies.

In the present study, we included patients with depression with different degrees of severity ranging from remission to severe. The within-group analysis showed that busyness-LL was linked to the severity of depression. Furthermore, busyness-LL showed a stronger link with the severity of pleasure loss than of depressive mood. The findings are consistent with the fact that pleasure loss is associated with the dysfunction of the reward system, especially the dopaminergic system in the limbic region of the striatum [35]. Our observations in the left side of the limbic striatum are consistent with the finding that the dysfunction of the left nucleus accumbens is involved in the reward processing in MDD [36].

The alternation in busyness-LL in depression was not explained by the effects of medication. Busyness-LL showed a significant relationship to the severity of depression in the medicated group and the same trend in the unmedicated group, although the latter was not statistically significant. In contrast, the link between the DVR-SML and severity of depression was found only in the unmedicated group, but not in the medicated group. Thus, the DVR values obtained from the conventional ROI analysis may be more sensitive to medication than the texture feature values obtained from texture analysis. Moreover, the findings in this study on the effect of medication on the DVR values may explain the discrepancy between the results of the depression-focused PET studies [7, 9]. A study on unmedicated patients reported a decrease in the DVR values of a subregion in the striatum [9], however, this was not confirmed in a study on medicated patients [7]. Our results indicate that the texture features in DAT-SPECT provides useful information even in medicated patients.

Studies in healthy subjects have shown that dopamine function is linked to age, sex and season [33, 37, 38]. DVRs in healthy individuals in the bilateral sensorimotor regions of the striatum were found to decrease with age, which is in contrast to busyness-LL in the healthy group, which was not linked to age. The inconsistency in the findings may be attributed to the nature of texture features. Texture features depend on several factors such as the intensity histogram of the image and the relative position of the voxels. In more detail, texture features are calculated based on the probability of occurrence of each intensity value, the level of spatial rate of change of intensity, and the magnitude of the difference between adjacent intensity values. Thus, texture features could not necessarily show similar change to the mean uptake value even if the mean uptake value decreases with age. Sex differences in DAT binding have been reported [38, 39], we did not find significant sex differences in DVRs as well as busyness-LL in the healthy group. This is due to the fact that our sample was comprised of relatively older individuals (that is, approximately 40% of our healthy samples were aged 60 years or older), and that sex differences observed in younger individuals were reportedly not observed in individuals aged 60 years or older [39]. Although a previous study indicated that there is a link between season and dopamine synthesis in the presynaptic striatum in humans [37], we did not find significant effects of the season of scans on DVR values or busyness-LL in the patient group (Table S4). This may be because the impact of depression on busyness-LL is more pronounced than in seasons.

Recently, SPECT imaging has been criticized for producing low-resolution images in comparison with PET [9]. However, texture analysis can quantify not only the mean uptake values but also the image textures, such as “heterogeneity” [19]. In other words, texture analysis is a technique that enables us to extract and evaluate more information from images. In PET and SPECT studies that focused on the heterogeneity of dopamine distribution in the striatum in PD, the texture feature values had been shown to have a higher correlation with the severity of PD than the DVR values [19, 40]. Furthermore, it has been confirmed that texture feature values derived from low-resolution images retain a high correlation with disease severity, which was comparable to PET images [40]. This suggests that texture analysis may have a potential to compensate for the weakness of low-resolution SPECT images.

In this study, the texture feature values showed a significant relationship with the clinical indices of depression compared with the DVR values. The results suggest that spatial heterogeneity of dopamine distribution exists in depression as well as in normal aging and PD [22]. Although the heterogeneity of dopamine distribution in depression has not yet been elucidated, this may be supported by a hypothesis of dopaminergic dysfunction in depression [41]. The hypothesis is underpinned by the fact that depressive states are related with a decrease in the proportion of spontaneously active neurons as well as the frequency of burst firing [42] and that these changes are reversible [43]. Notably, the changes in busyness-LL in normal aging were contradictory to those in depression in the present study (Fig. S7). Studies had shown that age-related changes irreversibly cause decreased number of dopamine neurons [44, 45], whereas the activities in the remaining neurons showed a compensatory increase [46]. These findings may support a notion that the difference in the change in busyness-LL between depression and aging can be attributed to the difference in the anatomical distribution of dopaminergic neurons with reduced DAT as well as the difference in the compensatory mechanisms of remaining neurons; the mechanism is preserved in aging, but not in depression.

It is noteworthy that we investigated the effect of depression on DAT binding in patients who had already developed the illness. Since previous studies on DAT binding in depression have evaluated the differences between patients with depression and healthy controls, it was difficult to determine whether the altered DAT binding in depression reflects the cumulative changes that occur after the onset of depression or a temporary depressive state. In the present study, we showed that busyness-LL was different between the depressive and remission states. We also confirmed that this finding was observed in the course of depression in the same individuals. To our knowledge, this is the first study to have clearly demonstrated that depressive state itself could alter DAT binding.

However, this study has some limitations. First, the ligand for DAT-SPECT (i.e.,^123^I-FP-CIT) has an affinity for the serotonin transporter (SERT), although it has a significantly lower affinity than that for DAT [47]. However, the texture feature values obtained in this study were less sensitive to the effect of antidepressants that have a very high affinity for SERT. Second, patients were recruited from a single hospital, and were all Japanese. Therefore, care should be taken when generalizing the results in the present study. Third, our healthy samples were not matched for age, sex, and SPECT imaging conditions (scanner, number of gray levels, voxel size, etc.), and thus we could not conduct a direct comparison with the patient group. Finally, additional validation in the unmedicated group and BD group failed to show a significant relationship with the severity of depression. However, a similar trend was observed in the overall results in both groups, and the nonsignificant findings may be attributable to the low statistical power associated with the insufficient number of the subjects.

In conclusion, by applying texture analysis to DAT-SPECT, we were able to indicate that the texture feature values in DAT binding were linked to the severity of depression in patients with mood disorders. Our findings could not be attributed to the effect of medication or age. Furthermore, texture analysis may be more informative than the conventional methods in DAT-SPECT analysis. Our findings also imply that the texture features in DAT-SPECT may be useful as a state-marker of depression.

## Supplementary information

Supplementary Information
