# Supplementary material for: Dopamine dysfunction in depression: application of texture analysis to dopamine transporter single-photon emission computed tomography imaging
Source: Transl Psychiatry. 2022 Aug 3;12:309. doi: 10.1038/s41398-022-02080-z (PMC9349249; doi:10.1038/s41398-022-02080-z)
Supplement: Supplementary file 1 — Supplementary Information [file 41398_2022_2080_MOESM1_ESM.docx]

**Supplementary Information**

**Dopamine dysfunction in depression: Application of texture analysis to dopamine transporter single-photon emission computed tomography imaging**

Takehiro Tamura, M.D., Genichi Sugihara, M.D., Ph.D., Kyoji Okita, M.D., Ph.D., Yohei Mukai, M.D., Ph.D., Hiroshi Matsuda, M.D., Ph.D., Hiroki Shiwaku, M.D., Ph.D., Shunsuke Takagi, M.D., Ph.D., Hiromitsu Daisaki, Ph.D., Ukihide Tateishi, M.D., Ph.D., Hidehiko Takahashi, M.D., Ph.D.

**Contents**

**Supplementary Table**

**Table S1.** Acquisition parameters of MR images.

**Table S2.** Texture feature values and sex.

**Table S3.** Texture feature values and age.

**Table S4.** Seasonal effects on DVR values or busyness-LL.

**Supplementary Figures**

**Figure S1**. Busyness-LL and the severity of depression according to the medication status.

**Figure S2.** Busyness-LL and the severity of depression in MDD and BD.

**Figure S3.** Busyness-LL and the severity of depression in those who had no smoking history in the previous year.

**Figure S4.** Busyness-LL and the severity of depression in those who had no receiving ECT in the previous year.

**Figure S5.** DVR-SML and the severity of depression in MDD and BD.

**Figure S6.** Longitudinal changes (depression state [pre-treatment] and remission [post-treatment]) in DVR-SML within the same individual (N = 12).

**Figure S7.** Differences between aging and depression in relation to busyness-LL.

**Table S1.** Acquisition parameters of MR images.

| **Parameters** | |
| --- | --- |
| Repetition time | 1800 (msec) |
| Echo time | 2.42 (msec) |
| Field of view | 250 (mm) |
| Voxel dimension | 1 mm × 1 mm × 1 mm; 256 slices |

Data of the MR images acquired using magnetization-prepared rapid gradient-echo (MP-RAGE) sequence.

Abbreviations: MR, magnetic resonance

**Table S2.** Texture feature values and sex.

| **Texture feature value** | **Males**  **Mean (SD)** | **Females**  **Mean (SD)** | **p value** |
| --- | --- | --- | --- |
| Coarseness-LL | 0.023560503 (0.003656958) | 0.022666761 (0.002988718) | 0.39 |
| Coarseness-EL | 0.014511329 (0.0030299) | 0.014067693 (0.00169063) | 0.813 |
| Coarseness-SML | 0.026215225 (0.004283114) | 0.026539785 (0.003772328) | 0.94 |
| Coarseness-LR | 0.027464824 (0.003622255) | 0.025732759 (0.003117982) | 0.153 |
| Coarseness-ER | 0.015635609 (0.003713029) | 0.017754286 (0.003836247) | 0.067 |
| Coarseness-SMR | 0.026489203 (0.006106521) | 0.028392842 (0.003736947) | 0.39 |
| Contrast-LL | 0.110501687 (0.019985724) | 0.117377198 (0.015970547) | 0.074 |
| Contrast-EL | 0.081793054 (0.010855367) | 0.081838394 (0.012263787) | 0.989 |
| Contrast-SML | 0.097387668 (0.038528552) | 0.09167965 (0.0098395) | 0.327 |
| Contrast-LR | 0.122095522 (0.01119404) | 0.123381491 (0.009835519) | 0.789 |
| Contrast-ER* | 0.099877646 (0.024645683) | 0.079107231 (0.015704764) | 0.002* |
| Contrast-SMR | 0.110043025 (0.044355858) | 0.094500835 (0.021485183) | 0.555 |
| Busyness-LL | 0.019419422 (0.005646176) | 0.021010627 (0.004933105) | 0.236 |
| Busyness-EL | 0.049109109 (0.012526288) | 0.042557156 (0.007246489) | 0.057 |
| Busyness-SML | 0.032913402 (0.013126544) | 0.02365672 (0.003606502) | 0.008* |
| Busyness-LR | 0.017140921 (0.004057337) | 0.019272922 (0.004173781) | 0.047* |
| Busyness-ER | 0.050082365 (0.022040869) | 0.034136649 (0.011355502) | 0.009* |
| Busyness-SMR | 0.034149759 (0.01290961) | 0.022827226 (0.006038736) | 0.002* |

The Mann-Whitney U test was performed to evaluate the differences in texture feature values between males and females in healthy subjects.

*p < 0.05

Abbreviations: LL, limbic region in the left striatum; EL, executive region in the left striatum; SML, sensorimotor region in the left striatum; LR, limbic region in the right striatum; ER, executive region in the right striatum; SMR, sensorimotor region in the right striatum

**Table S3.** Texture feature values and age.

| **Texture feature value** | **p value** |
| --- | --- |
| Coarseness-LL | 0.01* |
| Coarseness-EL | 0.429 |
| Coarseness-SML | 0.281 |
| Coarseness-LR | <0.001* |
| Coarseness-ER | 0.113 |
| Coarseness-SMR | 0.436 |
| Contrast-LL | 0.261 |
| Contrast-EL | 0.264 |
| Contrast-SML | 0.154 |
| Contrast-LR | 0.437 |
| Contrast-ER | 0.494 |
| Contrast-SMR | 0.324 |
| Busyness-LL | 0.073 |
| Busyness-EL | 0.661 |
| Busyness-SML | 0.996 |
| Busyness-LR | 0.007* |
| Busyness-ER | 0.747 |
| Busyness-SMR | 0.838 |

Pearson’s correlation test was used to evaluate correlations between age and texture feature values in healthy subjects.

*p < 0.05

Abbreviations: LL, limbic region in the left striatum; EL, executive region in the left striatum; SML, sensorimotor region in the left striatum; LR, limbic region in the right striatum; ER, executive region in the right striatum; SMR, sensorimotor region in the right striatum

**Table S4.** Seasonal effects on DVR values and busyness-LL.

| **Values** | **Spring–summer**  **Mean (SD)** | **Fall–winter**  **Mean (SD)** | **p value** |
| --- | --- | --- | --- |
| DVR-LL | 3.16 (0.46) | 3.25 (0.69) | 0.695 |
| DVR-EL | 2.65 (0.39) | 2.80 (0.54) | 0.093 |
| DVR-SML | 2.35 (0.32) | 2.41 (0.47) | 0.477 |
| DVR-LR | 3.08 (0.42) | 3.18 (0.66) | 0.726 |
| DVR-ER | 2.78 (0.42) | 2.89 (0.55) | 0.239 |
| DVR-SMR | 2.32 (0.34) | 2.36 (0.43) | 0.425 |
| Busyness-LL | 0.142 (0.033) | 0.146 (0.04) | 0.623 |

The Mann–Whitney U test was performed to evaluate the differences in DVR values or busyness-LL between spring-summer (from March 21 to September 20) and fall-winter (from September 21 to March 20) in the patient group.

Abbreviations: DVR, Distribution Volume Ratio; LL, limbic region in the left striatum; EL, executive region in the left striatum; SML, sensorimotor region in the left striatum; LR, limbic region in the right striatum; ER, executive region in the right striatum; SMR, sensorimotor region in the right striatum


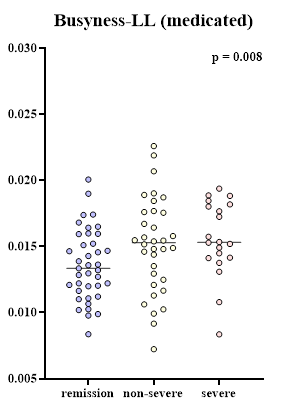

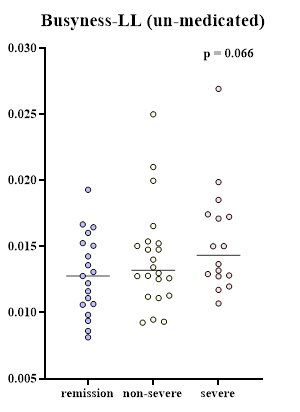


**Figure S1.**

Busyness-LL and the severity of depression according to medication status.

Abbreviations: LL, limbic region in the left striatum


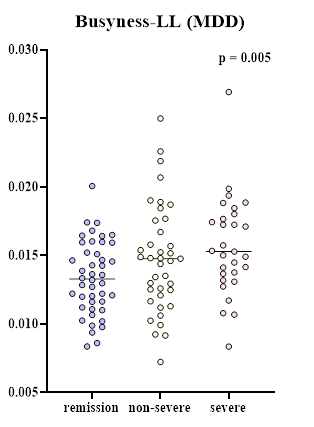

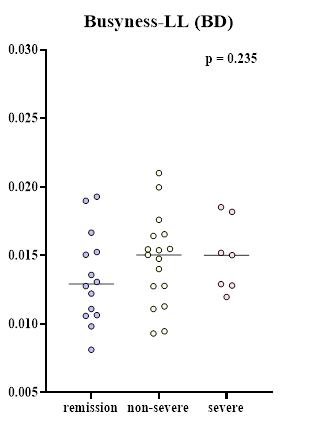


**Figure S2.**

Busyness-LL and the severity of depression.

Abbreviations: LL, limbic region in the left striatum


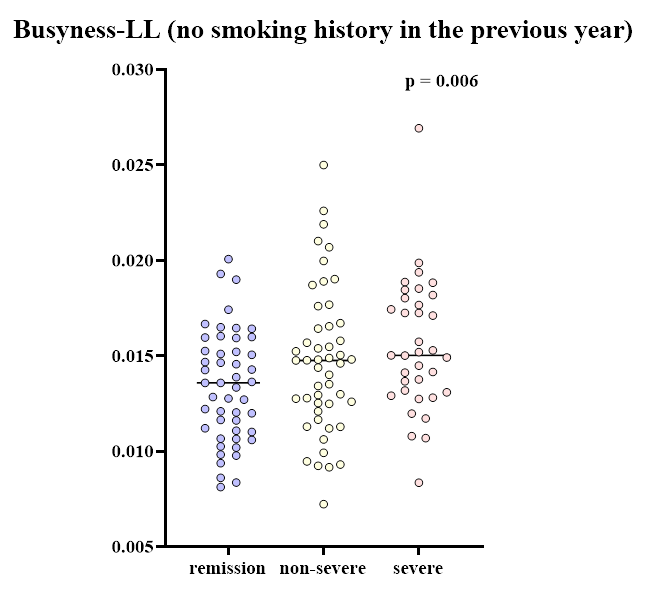


**Figure S3.**

Busyness-LL and the severity of depression in those who had no smoking history in the previous year.

Abbreviations: LL, limbic region in the left striatum


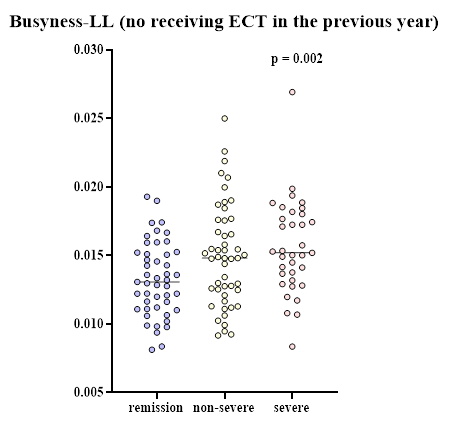


**Figure S4.**

Busyness-LL and the severity of depression in those who had no receiving ECT in the previous year.

Abbreviations: LL, limbic region in the left striatum; ECT, electroconvulsive therapy


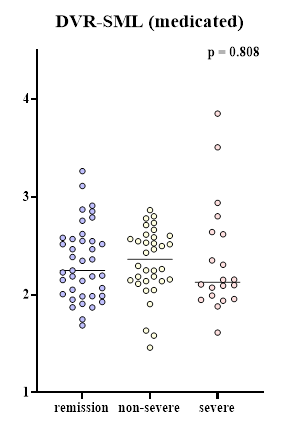

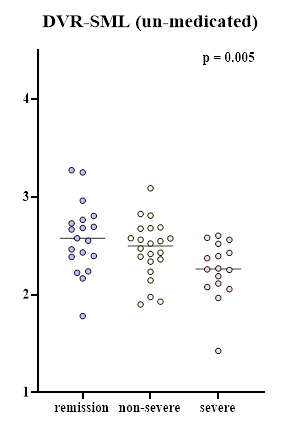


**Figure S5.**

DVR-SML and the severity of depression.

Abbreviations: DVR, distribution volume ratio; SML, sensorimotor region in the left striatum; SMR, sensorimotor region in the right striatum


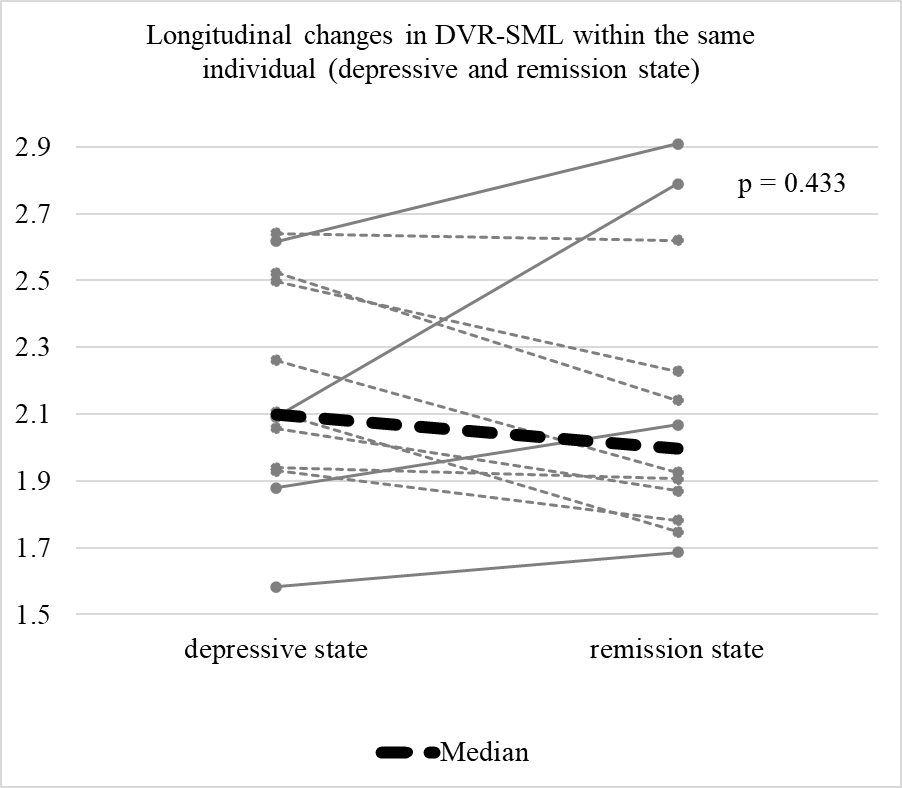


**Figure S6.** Longitudinal changes (depression state [pre-treatment] and remission [post-treatment]) in DVR-SML within the same individual (N = 12).

Abbreviations: DVR, distribution volume ratio; SML, sensorimotor region in the left striatum


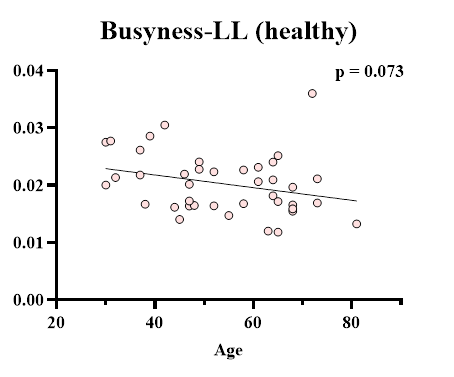

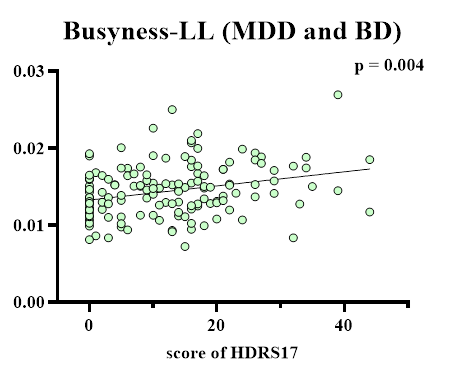


**Figure S7.** Differences between aging and depression in relation to busyness-LL.

Left: Busyness-LL and age.

Right: Busyness-LL and the severity of depression.

Pearson's correlation test was used to evaluate the correlation between busyness-LL and age in the healthy subjects and the patient group.

*p < 0.05

Abbreviations: LL, limbic region in the left striatum; MDD, major depressive disorder; BD, bipolar disorder
